# Simultaneous Co-transplantation for Highly Efficient Cell Therapy

**DOI:** 10.2174/011574888X359983250408105711

**Published:** 2025-04-22

**Authors:** Ji-Hee Choi, Mingu Ryu, Sung-Hwan Moon

**Affiliations:** 1 Department of Animal Science and Technology, Chung-Ang University, Anseong, Republic of Korea

**Keywords:** Cell therapy, co-transplantation, therapeutic efficacy, regenerative medicine, clinical application, incurable disease

## Abstract

Cell therapy involves transplantation of cells to replace damaged tissues and cells and is used in regenerative medicine. Since its introduction, numerous cell therapy modalities have been developed to treat various diseases, and cell therapy has shifted the paradigm of the treatment of degenerative and refractory diseases. However, it faces limitations in terms of long-term therapeutic effects and efficiency. To overcome these challenges, the concept of co-transplantation, which utilizes two different cell sources, has been proposed. Stem cell-based co-transplantation approaches have been extensively studied both experimentally and clinically for various diseases, including graft-versus-host disease (GVHD), infertility, acute liver failure (ALF), and myocardial infarction (MI). These have yielded improved transplantation efficiency and stability compared to single-cell transplantation methods. This review examines the development and effectiveness of co-transplantation through its application in four diseases. Additionally, it discusses the clinical applicability of co-transplantation, explores future research directions, and highlights its potential benefits.

## 1 INTRODUCTION

Since the introduction of cell therapy in 1889, various cell sources have been used over the years. Tissue regeneration using stem cells is currently actively researched because these cells have self-renewal and differentiation abilities [1, 2]. Stem cell-based cell therapy involves the direct injection of cells into the target tissue, offering the potential for regenerative effects. Immunomodulators secreted by stem cells are characterized by low immunogenicity and high regenerative potential and are well-suited for therapeutic applications. Notably, growth factors secreted by stem cells promote the proliferation and regeneration of damaged tissues. Cytokines and chemokines suppress inflammation at the site of injury and regulate immune responses, thereby maximizing regenerative effects. These molecules are critical in various physiological functions and regenerative mechanisms [3-5]. Clinical trials on stem cell therapies have been conducted for various diseases, including Parkinson’s disease, heart failure, graft-versus-host disease (GVHD), and diabetes. These therapies are particularly promising for treating degenerative disorders, cancer, and immune diseases [4, 6-9]. However, for stem cells to be utilized in cell replacement therapy or therapeutic biomaterials, several challenges must be overcome, including reducing immune rejection, improving differentiation efficiency, and addressing issues related to safety and regulatory approval [10, 11].

One of the biggest challenges is improving the survival rate of transplanted cells. Immune responses, along with oxidative stress, the absence of ligands, and the lack of growth factors, nutrients, and oxygen, lead to cell damage, ultimately decreasing cell survival rates and hindering long-term viability and functionality. As a result, the restoration of normal physiological functions in the transplanted tissue becomes unachievable [3, 12]. To enhance the post-transplantation cell survival rate, research is ongoing to evaluate the influence of various factors, including cell source, biomaterials, delivery route, time, and frequency of administration [3, 13, 14].

Co-transplantation aims to enhance the efficiency and functionality of cell transplantation by simultaneously or additionally supplying cytokine- and growth factor-secreting cells, which aid in the survival of transplanted cells. This approach increases the engraftment rate of cells, particularly when the amount of cells is low, by exploiting paracrine mechanisms, including anti-apoptotic, anti-inflammatory, and anti-oxidative effects, and by suppressing immune responses [15-17]. In this review, we discuss the applications and outcomes of co-transplantation in representative diseases, provide examples of its integration with tissue engineering technologies, evaluate its potential as a cell-based therapeutic strategy, and suggest further research directions.

## 2 CO-TRANSPLANTATION FOR GVHD

**Fig. (1):**
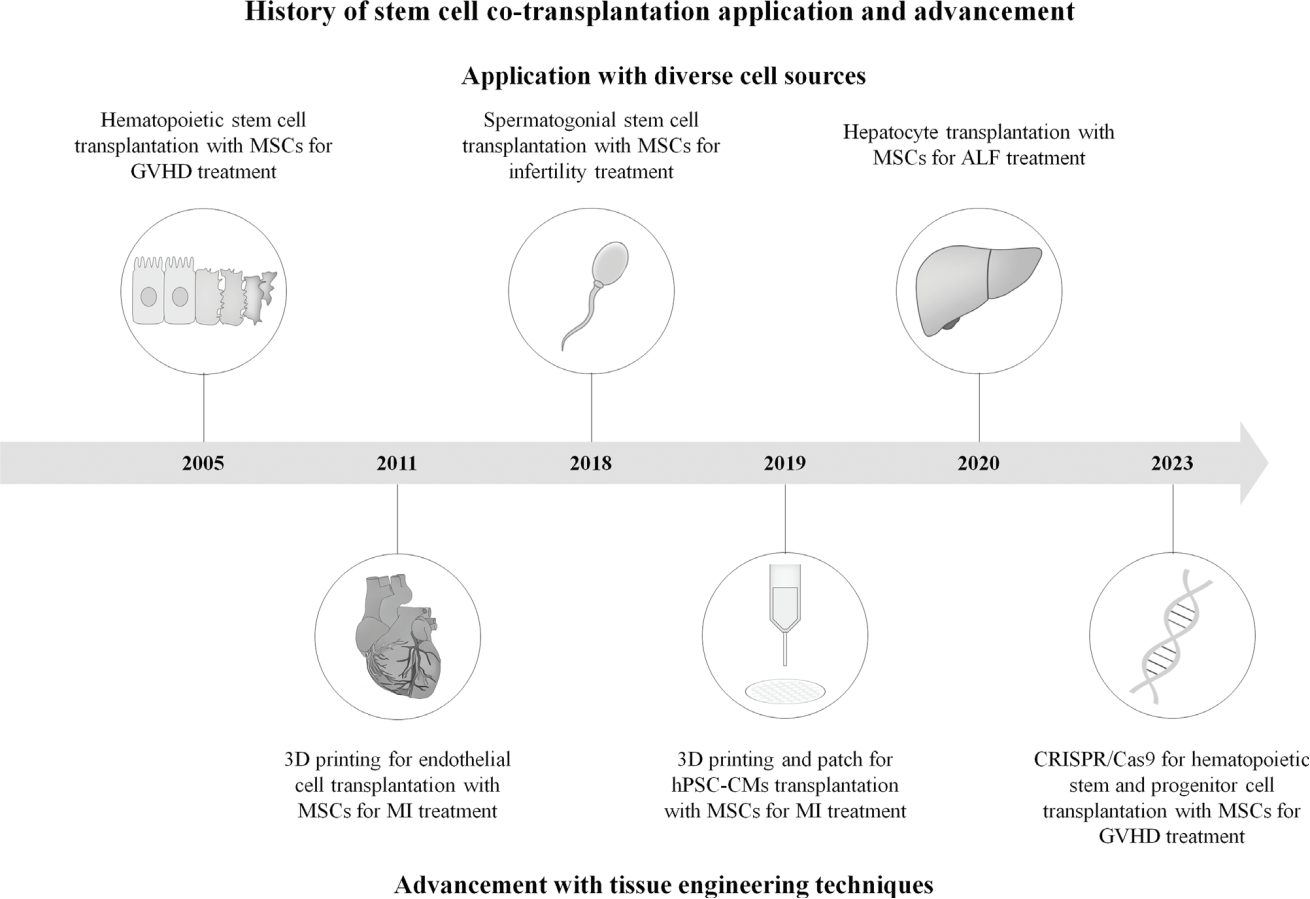
Critical events in stem cell co-transplantation research.

Among cell therapies utilizing co-transplantation, modalities for the treatment of GVHD following hematopoietic stem cell transplantation (HSCT) are the most extensively and consistently studied [18-25]. HSCT, recognized as the first clinical application of stem cell transplantation, is one of the few curative treatments available for malignant hematological diseases, including multiple myeloma, acute myeloid leukemia, and acute lymphocytic leukemia. Since the 1980s, HSCT has been widely implemented as a therapeutic intervention [21, 26]. However, GVHD, which arises from host antibody immune responses against transplanted T cells or aberrant immune reconstitution, is associated with high morbidity and mortality rates and often severe complications [27]. As this condition persists even after allogeneic HSCT, extensive research efforts are made to identify and mitigate risk factors to reduce immune rejection and enhance therapeutic outcomes [28-33]. MSCs reduce immune rejection and alleviate complications through various immunomodulatory mechanisms. First, they suppress the proliferation and cytotoxicity of T cells and natural killer (NK) cells, which attack transplanted cells, thereby enhancing the survival of transplanted cells, mitigating excessive immune responses, and reducing tissue damage. Additionally, during HSCT and co-transplantation, MSCs secrete hematopoietic cytokines to promote hematopoietic reconstruction, thereby shortening recovery time post-transplantation [34-36]. Since the early 2000s, the co-transplantation of MSCs in HSCT has garnered significant research interest, with numerous preclinical and clinical studies aimed at developing robust therapies for immune-related disorders (Table **1**, Fig. **1**) [37-40]. As a result of these efforts, MSC-based products, such as Ryoncil^TM^ and Temcell^®^, have been approved for therapeutic use in conjunction with HSCT in several countries, and efficacy and safety studies are ongoing [41-44]. Recent studies have evaluated the use of MSCs to reduce GVHD incidence following allogeneic HSCT from HLA-mismatched donors and repeated administration of MSCs to prevent chronic GVHD [45, 46]. Standardization of MSC manufacturing processes and quality control is essential to ensure consistent therapeutic outcomes. Furthermore, elucidating the precise mechanisms by which MSCs modulate immune responses and promote tissue repair to establish optimal treatment protocols is necessary. Long-term follow-up and monitoring are necessary to evaluate the sustainability of therapeutic effects and thoroughly assess potential adverse effects.

## 3 CO-TRANSPLANTATION FOR INFERTILITY

For the treatment of infertility, it is crucial to restore the function of spermatogenesis through the self-renewal activity of male germline stem cells and maintain this functionality in the long term [47]. Spermatogonial stem cell transplantation (SSCT) was first demonstrated to restore fertility in an infertile mouse model in 1994 and since then has been recognized as a promising technology with potential applications in fertility restoration in pediatric cancer patients, the treatment of male infertility, the conservation of endangered species, and breeding and agriculture [48-50]. However, results from SSCT studies on mice have shown a low homing efficiency of 12%, which has prompted preclinical studies in various animal models to address these limitations [47, 51]. Efforts are underway to establish SSCT-based therapies in larger animals and to improve the survival and efficiency of transplanted spermatogonial stem cells in rodent models. Two recent studies have explored the co-transplantation of MSCs to overcome the low efficiency of SSCT (Table **1**) [52-59]. In 2018, Kadam *et al*. [57] conducted a study to overcome the low homing efficiency of spermatogonial stem cells *via* co-transplantation with MSCs (Fig. **1**). Using a busulfan-induced infertility mouse model, green fluorescent protein-transfected spermatogonial stem cells were transplanted along with red fluorescent protein-transfected mouse bone marrow MSCs. After three months, testicular analysis revealed significant increases in both testis size and the testis-to-body weight ratio. Furthermore, sperm formation showed an approximately twofold increase compared to after SSC-only transplantation, demonstrating an improvement in fertility restoration efficiency. In a 2019 follow-up study, the same research group analyzed the offspring resulting from the co-transplantation (Fig. **1**). Postmortem examination of green fluorescent protein-expressing offspring revealed no anatomical abnormalities, and no defects were observed in the reproductive organs [58]. Co-transplantation of SSCT and MSCs restores the testicular niche and improves the testicular environment, thereby enhancing sperm formation and reproductive efficiency. MSCs play a crucial role by secreting growth factors and cytokines that promote sperm survival and proliferation while suppressing inflammation. GFP- and RFP-transfected cells have been used in a study to precisely track the integration and localization of the transplanted cell types, demonstrating the positive effects of co-transplantation on factors influencing reproductive capacity. However, the exact mechanisms underlying testicular niche recovery and improvement in reproductive efficiency remain unclear. Although the anatomical integrity of the offspring has been analyzed, further investigation into the long-term stability and fertility of subsequent generations has not been conducted. Moreover, follow-up studies examining outcomes under varying transplantation conditions or those evaluating the feasibility of this approach in humans are lacking. Additional research with diverse experimental designs and comprehensive long-term safety assessments is required to advance its clinical application.

## 4 CO-TRANSPLANTATION FOR ACUTE LIVER FAILURE (ALF)

ALF is characterized by the rapid onset of liver dysfunction in patients without prior liver disease, leading to coagulopathy and hepatic encephalopathy [60, 61]. ALF can be caused by drug toxicity, hepatic viral infections, or idiopathic factors, and its effects are not limited to the liver, as it also affects other organ systems, such as the kidneys, brain, pancreas, and cardiovascular system, often resulting in infectious complications [60, 62-64]. ALF has a mortality rate of approximately 30%, and in severe cases, liver transplantation is required. ALF accounts for approximately 8% of all liver transplants [63, 65, 66]. Several clinical trials have focused on restoring liver function through hepatocyte transplantation, and recently, improvements in liver function through the co-transplantation of hepatocytes and MSCs have been reported (Table **1**) [67-72]. In 2020, Cheng-Maw *et al*. [71] investigated the co-transplantation of hepatocytes and MSCs in a rat model of ALF (Fig. **1**). Animals were divided into a hepatocyte-only transplantation group and a hepatocyte-MSC co-transplantation group. The study revealed that co-transplantation increased albumin secretion and the expression of liver-specific genes, demonstrating improved hepatocyte function. Additionally, MSCs inhibited hepatocyte apoptosis and inflammatory responses while promoting hepatocyte proliferation, thus contributing to the protection of the damaged liver. The study further demonstrated long-term survival for more than six months in the co-transplantation group, confirming the safety and efficacy of the approach. Interestingly, the study revealed that MSCs not only supported normal liver function through paracrine effects but also differentiated into hepatocyte-like cells, directly contributing to the treatment of ALF. This finding suggested that sub-cells, previously considered to primarily support the function of main cells in co-transplantation, possess the potential to differentiate into the main cell type, thereby providing direct therapeutic benefits. Research on co-transplantation in ALF has recently gained traction, and while their results are still limited, studies utilizing various cell sources are actively underway. In 2019, a study demonstrated a short-term improvement in liver function by combining umbilical cord-derived mesenchymal stem cell (UC-MSC) transplantation with plasma exchange therapy in patients with ALF. Additionally, a clinical trial involving 150 patients with acute-on-chronic liver failure to evaluate the effects of MSC transplantation in combination with standard medical therapy is underway in Beijing 302 Hospital to analyze the optimal dosing frequency and treatment duration [73, 74]. These studies suggest the therapeutic potential of co-transplantation with MSCs in ALF, highlighting their role in immunomodulation and liver function recovery. Furthermore, integrating cell-based therapy with conventional treatment strategies is expected to present a new paradigm in the management of ALF.

## 5 CO-TRANSPLANTATION FOR MYOCARDIAL INFARCTION (MI)

MI is caused by vascular wall inflammation due to coronary artery disease and is a leading cause of death worldwide [75]. MI results in permanent damage to the heart muscle, leading to heart failure, arrhythmias, and various complications, rendering it a primary cause of heart failure [76, 77]. Cardiomyocytes have a limited capacity for self-regeneration and proliferation, and because of structural limitations, large-scale cell transplantation is challenging, leaving heart transplantation as the only definitive treatment for MI [78-80]. Since the early 2000s, transplantation methods using stem cells, such as bone marrow-derived mononuclear cells and bone marrow-derived MSCs (BM-MSCs), have been explored. In the 2010s, several studies were conducted on the combination of co-transplantation with 3D printing technologies for MI treatment (Table **1**) [81-87]. These studies improved MI treatment efficacy by enhancing the efficiency of cardiomyocyte transplantation, promoting functional regeneration, and increasing neovascularization. Additionally, ECs are the most abundant cell type in the adult heart and vascular system and play a crucial role in promoting neovascularization, forming vascular barriers, and facilitating blood supply in the heart [88, 89]. ECs regulate genes involved in cardiomyocyte growth and cardiac regeneration, highlighting their potential for use in cardiovascular therapies. Therefore, numerous studies have investigated the use of ECs in treating MI [90-92].

In 2019, Park *et al*. conducted co-transplantation of cardiomyocytes and MSCs to treat MI [86]. Human MSCs (hMSCs) in patch form, known as hMSC patches, have been developed to enhance cell survival post-transplantation. *In vitro* analysis demonstrated that more than 90% of cells survived after transplantation, with a time-dependent increase in paracrine factor release, contributing to vascular regeneration and cardiac function and regeneration. Experiments in MI model rats revealed that the co-transplantation of hiPSC-CMs and hMSC patches improved cardiac function, functional capillary formation, and cardiomyocyte maturity [86]. This study provided evidence of structural and functional cardiac regeneration through the co-transplantation of hiPSC-CMs and hMSC patches *in vitro* and *in vivo*. Additionally, the study demonstrated the long-term efficacy of this approach, with successful outcomes lasting beyond 8 weeks, indicating it is a promising and effective cell therapy strategy for MI. In 2022, Kim *et al*. conducted a study on the co-transplantation of endothelial cells and MSCs for treating MI [87]. Therapeutic neovascularization can be divided into two processes: angiogenesis and vasculogenesis, both of which are essential for the repair of damaged cardiovascular systems [93, 94]. While angiogenesis occurs during both embryonic development and adult life, vasculogenesis primarily occurs during embryonic development, with few exceptions in specific physiological contexts [95]. Given this distinction, a co-transplantation strategy was designed to promote neovascularization by using hiPSC-ECs for vasculogenesis and engineered human mesenchymal stem cells secreting stromal-derived factor-1α (SDF-eMSCs) in a 3D patch form to support angiogenesis. *In vitro* experiments demonstrated that the hiPSC-ECs improved cardiac function as evidenced by an increase in the ejection fraction and fractional shortening, whereas the hMSCs enhanced the expression of genes and cytokines involved in EC function and angiogenesis. Co-transplantation in an MI model confirmed the therapeutic potential of this approach, as it resulted in increased expression of cardiac markers, reduced cardiac fibrosis, and significant neovascularization *in vivo*. These findings highlight the contribution of this strategy to cardiac repair and vascular regeneration, demonstrating its promise as an effective therapeutic approach for cardiovascular diseases. In 2011, Gaebel *et al*. [96] evaluated the function of a cardiac patch designed to promote neovascularization in an MI model using laser printing to create a scaffold combining ECs and MSCs (Fig. **1**). Using the laser-induced forward transfer technique, human umbilical vein ECs (HUVECs) and hMSCs were printed onto a polyester urethane urea cardiac patch in a grid pattern. Human umbilical vein ECs and hMSCs co-cultured for eight days accelerated vascular formation *in vitro*. Upon transplantation into an MI rat model, the cardiac patch increased capillary density, enhanced neovascularization, and improved functional vascular connections, leading to a significant improvement in cardiac function. This study demonstrated that processing the polyester urethane urea material into an interconnected micropore structure enhanced cell-cell interactions, whereas the laser-induced forward transfer-based printing technique augmented the vascular formation capabilities of ECs and MSCs. By integrating biomaterials and 3D printing technology into co-transplantation, the natural vascular structure was mimicked, enhancing cellular interactions and leading to increased and stabilized neovascularization, which contributed to the improvement of MI outcomes.

## 6 ADVANCEMENT IN CO-TRANSPLANTATION

The introduction of the concept of tissue engineering in the late 1980s led to various attempts to replace damaged tissues and restore their function across multiple fields [97, 98]. Since then, tissue engineering has evolved significantly, including advancements in stem cell technology, 3D printing, biomaterials, and gene editing to address the limitations of organ transplantation. Research and clinical trials to evaluate the efficacy and safety of these technologies are ongoing [99-101]. The improved transplantation efficiency observed with co-transplantation in various diseases has encouraged further studies. Recently, several studies have explored the integration of tissue engineering technologies into co-transplantation strategies to improve transplantation outcomes (Table **1**). Since the development of the CRISPR/Cas9 gene-editing tool, extensive research has been conducted on HSPCs to explore the therapeutic potential of gene-edited autologous transplantation [102, 103]. However, issues, including DNA damage responses and p53 activation following gene editing, which inhibit cell proliferation, impair engraftment, and increase cell death, necessitate further investigation to resolve them and improve transplantation efficiency [104-107]. In 2023, Crippa *et al*. [108] combined co-transplantation with gene-editing techniques to optimize the efficiency of CRISPR-Cas9 gene-edited HSPCs (GE-HSPCs) through the development of a two-dimensional co-culture system with MSCs and evaluated its functionality (Fig. **1**). The effects of GE-HSPCs cultured alone were compared with those of GE-HSPCs co-cultured with MSCs, showing that co-culturing significantly enhanced cell proliferation, colony formation, and increased the population of primitive HSPCs. To optimize the numbers of total cells and primitive HSPCs, the researchers adjusted the culture environment and duration. To maximize hematopoietic function, a two-dimensional co-culture system was developed utilizing MSCs and human umbilical vein ECs, allowing for the cultivation of optimal GE-HSPCs for transplantation. The GE-HSPCs were transplanted into an immunodeficient mouse model. Post-transplantation, peripheral blood analysis revealed that the co-transplantation approach led to an increase in the engraftment rate and significant increases in CD34+ and CD45+ cell numbers, demonstrating a time-dependent improvement up to 16 weeks, confirming the stability and efficacy of this approach.

## CONCLUSION

In the above four representative diseases requiring cell transplantation for therapeutic purposes, co-transplantation has demonstrated synergistic effects, with increased therapeutic efficacy, survival rates, and immune suppression when compared with the transplantation of only target cells. Preclinical and clinical studies have investigated the potential of co-transplantation to enhance the efficiency of various cell sources, improve tissue function recovery, and promote long-term survival. However, significant challenges remain to be overcome before co-transplantation can be widely applied as a cell therapy for human diseases in clinical settings. This review aims to provide new insights by exploring previously under-researched aspects, particularly the interactions between co-transplanted cell types and the resulting synergistic effects on therapeutic outcomes. Unlike other reviews that have predominantly focused on single-cell transplantation, our review critically evaluates the enhanced value of stem cell co-transplantation within the framework of immune modulation and tissue repair mechanisms. Despite the increased efficiency, issues, such as low engraftment and survival rates, persist, necessitating the establishment of more reliable therapeutic approaches. The lack of standardization and guidelines in stem cell therapies has delayed the clinical application of stem cell products. To address this issue, organizations, such as the FDA and the International Society for Stem Cell Research, are continuously establishing regulations and clinical trial guidelines to optimize grafts and improve the safety of regenerative therapies through quality control measures for cell resources [109]. This emphasizes the need for technological standardization to enhance the success rates of cell transplantation and develop more reliable therapeutic strategies. In co-transplantation studies, MSCs have long been the primary cell source utilized, with the potential of other cell types remaining largely unexplored. However, recent findings suggest that co-transplanting olfactory ensheathing cells alongside neural stem cells can exert synergistic effects, highlighting the promising potential of exploring diverse cell combinations [110]. The use of two cell sources in co-transplantation also requires a deeper understanding of the interactions between cells and immune mechanisms to ensure long-term safety. Therefore, future research should focus on identifying a broader range of cell types, evaluating their therapeutic potential, and assessing their interactions within co-transplantation environments. It is crucial to monitor the long-term survival and functionality of transplanted cells, as well as the potential complications and immune responses that may arise post-transplantation. These advancements hold potential as an alternative to organ transplantation and offer a pathway to personalized treatments within regenerative medicine, thereby facilitating groundbreaking progress in the treatment of intractable diseases. In summary, such progress is expected to drive transformative advancements in managing refractory conditions and redefining the possibilities of regenerative medicine.

## Figures and Tables

**Table 1:** Methods and outcomes of clinical and non-clinical studies on co-transplantation.

**Disease**	**Application**	**Transplantation Method**			**Therapeutic Effects**	**References**
		**Main Cell Source**	**Sub Cell Source**	**Technique**		
Graft-versus- host disease (GVHD)	Clinical	Hematopoietic stem cell	MSCs	-	34/46 patients alive	[19]
			MSCs	-	6/8 patients alive	[20]
			MSCs	-	10/14 patients alive	[23]
			MSCs	-	4/7 patients alive	[18]
			BM-MSCs	-	4/4 patients alive	[24]
			MSCs	-	11/25 patients alive	[25]
			MSCs	-	10/11 patients alive	[21]
			CB	-	8/11 patients alive	
			BM-MSCs	-	17/28 patients alive	[39]
		CB	MSCs	-	5/5 patients alive	[33]
	Preclinical	Hematopoietic stem and progenitor cell	MSCs	CRISPR/Cas9	Hematological reconstitution ↑	[108]
Acute liver failure (ALF)	Preclinical	Hepatocyte	LMSCs	-	Hepatocyte cell engraftment ↑	[72]
			BM-MSCs	-	Hepatocyte-like cell ↑	[71]
Infertility	Preclinical	Spermatogonial stem cell	MSCs	-	Spermatozoon formation ↑	[57]
				-	Tubular fertility index ↑	[58]
Myocardial infarction (MI)	Preclinical	Endothelial cell	MSCs	3D printing with patch	Vascular connection ↑	[96]
		hPSC-CMs			Capillaries number ↑	[86]
		hPSC-ECs			Neovascularization	[87]

**Note:** The upward arrow (↑) indicates an increase in the corresponding parameter.

## LIST OF ABBREVIATIONS

ALF: Acute Liver Failure
BM-MNCs: Bone Marrow Mononuclear Cells
EC: Endothelial Cell
GVHD: Graft-Versus-host Disease
hiPSC-CMs: Human-induced Pluripotent Stem Cell-derived Cardiomyocytes
hiPSC-ECs: Human-induced Pluripotent Stem Cell-derived Endothelial Cells
HSCT: Hematopoietic Stem Cell Transplantation
HSPC: Hematopoietic Stem and Progenitor Cell
MI: Myocardial Infarction
MSC: Mesenchymal Stem Cell
SSCT: Spermatogonial Stem Cell Transplantation
